# Carbon Nanotube Supported Molybdenum Carbide as Robust Electrocatalyst for Efficient Hydrogen Evolution Reaction

**DOI:** 10.3390/molecules28010192

**Published:** 2022-12-26

**Authors:** Yunjie Huang, Yaqi Bao, Tieqi Huang, Chengzhi Hu, Haiou Qiu, Hongtao Liu

**Affiliations:** 1 Faculty of Materials Science and Chemistry, China University of Geosciences, Wuhan 430074, China; 2 College of Chemistry and Chemical Engineering, Central South University, Changsha 410083, China

**Keywords:** molybdenum carbide, carbon nanotube, electrocatalyst, hydrogen evolution reaction

## Abstract

Molybdenum carbide is considered to be one of the most competitive catalysts for hydrogen evolution reaction (HER) regarding its high catalytic activity and superior corrosion resistance. But the low electrical conductivity and poor interfacial contact with the current collector greatly inhibit its practical application capability. Herein, carbon nanotube (CNT) supported molybdenum carbide was assembled via electrostatic adsorption combined with complex bonding. The N-doped molybdenum carbide nanocrystals were uniformly anchored on the surfaces of amino CNTs, which depressed the agglomeration of nanoparticles while strengthening the migration of electrons. The optimized catalyst (250-800-2h) showed exceptional electrocatalytic performance towards HER under both acidic and alkaline conditions. Especially in 0.5 M H_2_SO_4_ solution, the 250-800-2h catalyst exhibited a low overpotential of 136 mV at a current density of 10 mA/cm^2^ (η_10_) with the Tafel slope of 49.9 mV dec^−1^, and the overpotential only increased 8 mV after 20,000 cycles of stability test. The active corrosive experiment revealed that more exposure to high-activity γ-Mo_2_N promoted the specific mass activity of Mo, thus, maintaining the catalytic durability of the catalyst.

## 1. Introduction

Hydrogen is one of the most promising renewable and clean sources owing to its environmental friendliness and sufficient supply from water splitting driven by renewable energy, such as solar and wind energy [1,2,3]. The development of low-cost and high-performance catalysts to replace platinum is one of the preconditions for large-scale production of hydrogen from water electrolysis [4,5]. Molybdenum carbide is considered to be one of the most competitive catalysts for hydrogen evolution reaction (HER) regarding its d-band structure is similar to platinum and its high abundance of molybdenum in the earth’s crust that is 2~3 orders of magnitude higher than that of platinum [6,7].

To improve the catalytic performance of molybdenum carbide, various strategies, including morphological and compositional modification [8,9], defect engineering [10,11,12], crystallite regulation [13], heterojunction construction [14,15,16], and carbon-supported structure design [17,18,19] have been proposed. Among them, the carbon-supported molybdenum carbide showed a great potential to maximize the catalytic capability of molybdenum carbide by optimizing the carbon interface. The effective interfacial contact between molybdenum carbide and carbon substrate facilitates electronic migrations from molybdenum to carbon, thus, weakening H-Mo binding, which is supposed to be beneficial to water splitting [6,20]. Furthermore, the supporting effect of carbon substrate is conducive to anchoring small-sized molybdenum carbide, reducing their agglomeration. Roughly there are two types of carbon-supported molybdenum carbide construction. One is supporting molybdenum carbide onto the prepared carbon [17,18,19]; another is the in-situ formed carbon-supported molybdenum carbide [21,22,23,24,25,26]. The main advantage of the former is that the preparation process is relatively simple and controllable, and the used carbon substrate is highly graphitized. But this fabrication usually results in poor contact between molybdenum carbide and carbon. By contrast, the in-site construction can create good interfacial contact, facilitating the transfer of electrons, though the graphitization degree of carbon is a little bit low. To enhance the interaction between molybdenum carbide and carbon substrate, it is necessary to functionalize either molybdenum or carbon sources for better matching the pairs. Fu et al. [25] modified reduced graphene oxide (rGO) with polyethyleneimine and formed a positively charged carbon surface, which could attract the negatively charged PMo_12_O_40_ ions and realized the self-assembly of molybdenum species on a carbon support. As a result, the small-sized molybdenum carbide nanoparticles were well dispersed on the surface of rGO support without evident agglomeration. In another study, Qamar et al. [26] used oxalate to coordinate molybdenum, and the formed Mo-oxalate complex could effectively assemble onto the surface of a carbon nanotube (CNT). The obtained CNT-supported molybdenum carbide electrocatalyst showed superior performance towards HER.

In this work, carbon-supported molybdenum carbide has been constructed by a combination of amino CNTs and molybdate. Due to the strong protonation of -NH_2_, the aqueous amino CNTs tended to be positively charged, making molybdate anions spontaneously anchored onto the surface of CNTs by electrostatic adsorption [27,28]. During the subsequent heat treatment process, the molybdenum carbide nanocrystals were gradually developed and uniformly dispersed on the surface of CNTs. The as-prepared CNT-supported molybdenum carbide exhibited good interfacial electronic transfer capability, and the targeted catalyst showed excellent electrocatalytic performance toward HER in both acidic and basic solutions. This study provides a simple and reasonable method to construct good interfacial contact between molybdenum carbide and carbon support, thereby greatly promoting the property of the catalyst for HER.

## 2. Experimental

### 2.1. Reagents

Ammonium molybdate tetrahydrate, ethanol, potassium hydroxide, hydrochloric acid, and sulfuric acid were all analytical grade and obtained from Sinopharm Chemical Reagent Co. (Shanghai, China). Nafion^®^ (5 wt.%), amino muti-walled CNTs, and Pt/C (20 wt.%) were acquired from Alfa Aesar, Aladdin, and Johnson Matthey, respectively. All chemicals were used as received without any additional treatment.

### 2.2. Preparation

250 mg of amino multi-walled CNTs were added to a beaker containing 80 mL of water. After ultrasonic dispersion for 30 min, ammonium molybdate aqueous solution (250 mg ammonium molybdate tetrahydrate dissolved in 20 mL of water) was added drop by drop. The suspension was stirred to form a paste and then transferred into a vacuum oven to dry at 60 °C for 10 h. The obtained black powder was put into a tube furnace filled with CH_4_/H_2_ (20/80 mL/min) mixing gas and heated for 2 h at 800 °C. The collected product was labeled as 250-800-2h (250 mg ammonium molybdate tetrahydrate, 800 °C, 2 h). To study the influence of preparation conditions on the catalyst, the mass of ammonium molybdate tetrahydrate, calcination temperature, and calcination time were changed. The obtained contrast samples include 250-600-2h, 250-700-2h, 250-900-2h, 250-800-1h, 250-800-4h, 150-800-2h, 200-800-2h, and 300-800-2h. In addition, the sample (250-800-2h) was treated with 0.5 M HCl for 72 h to reveal the acid corrosion effect.

### 2.3. Characterization

X-ray diffraction (XRD) patterns were recorded over a D8-Focus diffractometer operated using Cu K radiation of 0.154 nm wavelength. The scanning step and scanning speed were 0.01° and 0.05°/s, respectively. Scanning electron microscope (SEM) was SU8010 mode of Hitachi with the acceleration voltage of 0.1–30 kV and equipped with X-ray Energy Dispersion Spectrometer (EDS). X-ray photoelectron spectroscopy (XPS) was performed on a K-Alpha model of Thermo Fisher operated using Al K_α_ radiation. The signal binding energy was calibrated against the C1s peak (284.64 eV) of adventitious carbon. The thermogravimetric analyzer was STA409PC mode of Netzsch Instrument. The test was carried out in an oxygen atmosphere from room temperature to 800 °C at a rate of 5 °C/min.

Electrochemical tests were carried out using a standard three-electrode potentiostat/galvanostat system (Interface 1000, Gamry, Warminster, PA, USA). The reference and counter electrodes were a saturated calomel electrode (SCE) and a graphite rod, respectively. Before the experiment, the potential of SCE relative to a reversible hydrogen electrode (RHE) was calibrated in 0.5 M H_2_SO_4_ solution and 1 M KOH solution (Figure S1), respectively. The preparation method of the working electrode was as follows. First, 5.0 mg of catalyst was placed into a 1.5 mL centrifuge tube. Then 800 μL of ultra-pure water, 150 μL of anhydrous ethanol, and 50 μL of 5% Nafion solution were added successively. After ultrasonic dispersion of the centrifuge tube for 40 min, 10 μL of dispersed droplets were removed from the centrifuge tube to the surface of a glassy carbon electrode with a diameter of 3 mm and left for drying at room temperature. The polarization curves were obtained by linear sweep voltammetry (LSV) with IR compensation at a 2 mV/s scan rate. The geometric current density was calculated by dividing the measured current by the geometric area of the glassy carbon electrode. Electrochemical impedance (EIS) tests were performed at 150 mV with a potential amplitude of 5 mV in frequencies from 10^6^ to 0.1 Hz.

Cyclic voltammetry (CV) was conducted to check the electrochemical double-layer capacitance (C_dl_) of catalysts at non-Faradaic overpotentials, which could be applied to estimate the electrochemically active surface areas. CV tests were performed at scan rates of 50–300 mV/s in the range of 0 to 0.3 V. A linear trend was obtained by plotting the difference in current density between the anodic and cathodic sweeps at 0.15 V against the scan rate. The slope of the fitting line is equal to twice C_dl_. HER durability tests were conducted in 0.5 M H_2_SO_4_ and 1 M KOH solution in the potential range of −0.3–0.3 V at a scan rate of 100 mV s^−1^ for different cycles, respectively. LSV before and after the potential cycling were carried out for HER activity comparison. A long-term stability test was also conducted by performing chronoamperometry measurement at −150 mV in 0.5 M H_2_SO_4_ solution.

## 3. Results and Discussion

**Figure 1:**
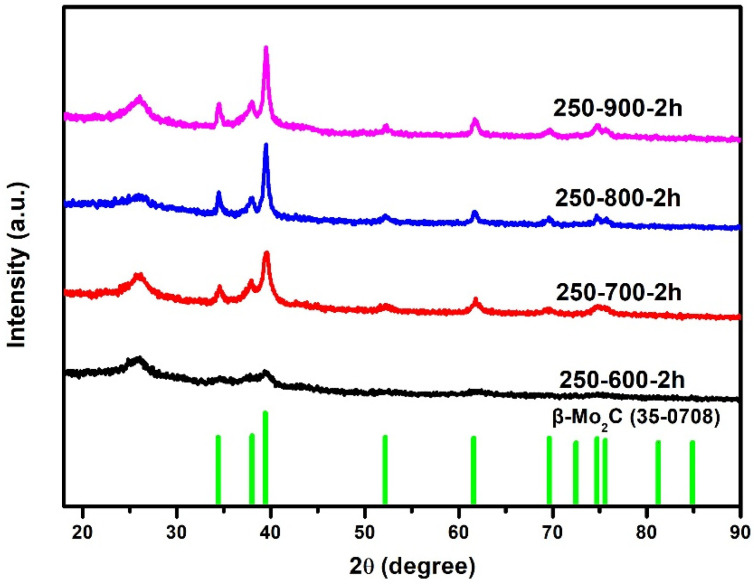
XRD patterns of catalysts with different calcination temperatures.

Figure 1 shows the XRD patterns of the products obtained at different calcination temperatures. By comparing with a standard card of β-Mo_2_C, it can be seen that the products calcined at 600–900 °C contain both β-Mo_2_C and graphite-carbon phases. The diffraction peak at 26° correlates to the (002) crystal plane of graphite-carbon, while the peaks at 34.35°, 38.98°, 39.39°, 52.12°, 61.53°, and 69.56° can be attributed to (100), (002), (101), (102), (110), and (103) crystal plane of β-Mo_2_C, respectively. It is also observed that the relative intensity of the β-Mo_2_C diffraction peak increases with the calcination temperature, indicating that high temperature is conducive to the formation of β-Mo_2_C with high crystallinity.

**Figure 2:**
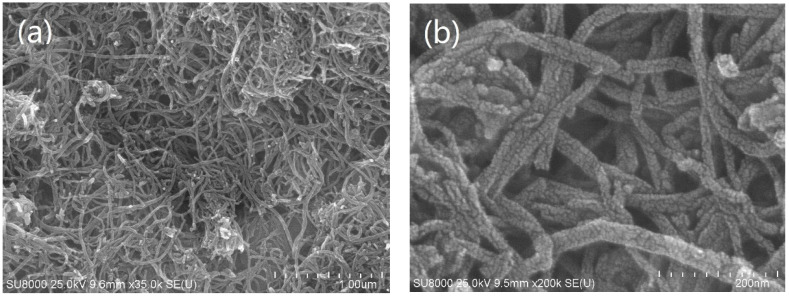
SEM images of the 250-800-2h catalyst with different magnifications: (**a**) 35,000 times, (**b**) 200,000 times.

As a representative catalyst, the morphology of the 250-800-2h sample was observed by SEM with different magnifications (Figure 2a,b). Apparently, the catalyst is composed of long nanotubes, and the surfaces of these nanotubes are covered with a layer of small particles. Combined with the XRD results (Figure 1) and elemental distributions (Figure S2), these small particles are β-Mo_2_C nanocrystals, which are well dispersed on the surfaces of the CNTs. The mean size of the particles is ~7 nm.

**Figure 3:**
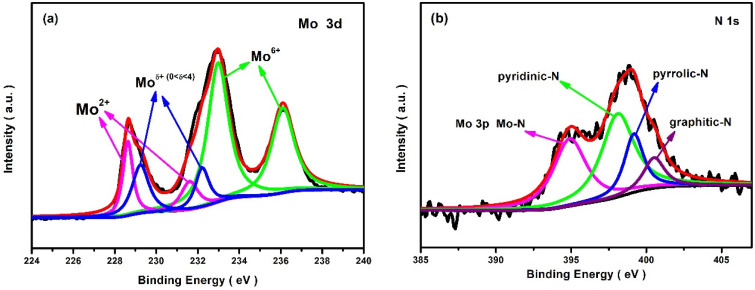
XPS spectra of the 250-800-2h catalyst, (**a**) Mo 3d and (**b**) N1s.

Figure 3 shows the XPS results of the 250-800-2h catalyst. On the Mo 3d XPS spectrum, Mo^2+^, Mo^δ+^ (0 < δ < 4+), and Mo^6+^ can be identified from their characteristic peaks corresponding to 3d_5/2_ and 3d_3/2_ electronic states, respectively [15,28]. On the N 1s XPS spectrum, the peak at 394.9 eV belongs to the Mo-N bond. But the characteristic diffraction peak of molybdenum nitride is not observed in the XRD patterns of Figure 1, hinting at its non-crystalline structure. Besides the Mo-N bond, there are some atomic N doped into the carbon crystal lattice, which is confirmed by the peaks at 398.1 eV, 399.2 eV, and 400.5 eV corresponding to pyridine-N, pyrrole-N, and graphite-N, respectively. These XPS results reveal that N atoms in the precursors have been involved in the chemical reactions associated with Mo and C. Actually, N doping in molybdenum carbide or carbon matrix can effectively regulate the electronic distributions around the active center, thereby optimizing the electrocatalytic activity of the catalyst [6,23,29,30].

**Figure 4:**
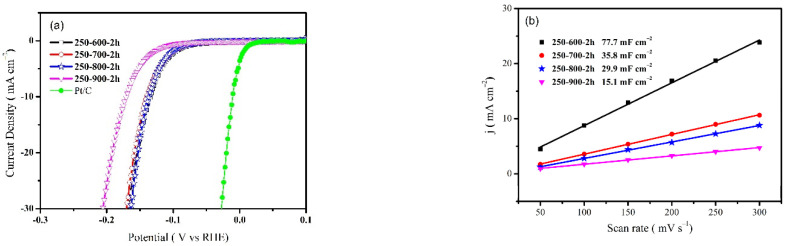

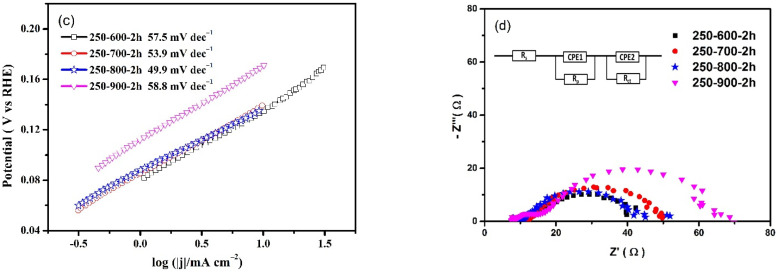
(**a**) polarization curves, (**b**) capacitance plots (at 0.15 V), (**c**) Tafel plots, and (**d**) Nyquist plots (η = 250 mV) of different catalysts in 0.5 M H_2_SO_4_ solution.

Figure 4a shows the LSV behaviors of the catalysts with different calcination temperatures in 0.5 M H_2_SO_4_ solution. The Pt/C catalyst was also tested as the contrast. Evidently, the 250-900-2h catalyst has a more negative HER potential than the other catalysts, implying relatively weaker catalytic activity. The 250-800-2h catalyst exhibits the best HER activity, and its overpotential at 10 mA cm^−2^ (η_10_) is only 137 mV. To further disclose the specific area activity of the catalysts, the electrochemical active areas were estimated by measuring the double-layer capacitance at non-faradic intervals. It can be seen in Figure 4b that the electrochemically active area decreases with the increase in calcination temperature. Considering its most positive HER potential with a relatively smaller active area, the 250-800-2h catalyst shows the highest specific area activity [31]. During the HER process in an acid solution, the desorption of hydrogen is supposed to be the rate-determining step (RDS). The kinetics of the RDS reaction can be measured by the Tafel plot. Figure 4c compares the Tafel slopes of the catalysts. The 250-800-2h catalyst also presents the smallest value of 49.9 mV dec^−1^, indicating its fastest kinetic rate. In addition, the electrochemical impedances were measured, and the corresponding Nyquist plots are shown in Figure 4d. The 250-800-2h sample exhibits the smallest charge-transfer barrier, suggesting its good electron transfer capability. Based on the above results, the 250-800-2h catalyst shows the highest catalytic activity due to the excellent charge-transfer kinetics.

The effects of calcination time and molybdate content under 800 °C on the catalytic activity of the catalyst were investigated synchronously. It can be seen in Figure S3 that the 250-800-2h catalyst is superior to the others, indicating that the proper calcination time is ~2 h. Too long may cause particle sintering, while too short often results in incomplete crystallization. Figure S4 reveals the optimal amount of molybdate precursor. For a certain amount of CNT substrate, the rational exposure of molybdenum active sites at the surface is very important. Too little molybdenum content cannot provide enough active sites to catalyze HER, while too much molybdenum loading can cause overlapping of active sites, thus, reducing the effective exposure of molybdenum active sites. Due to proper molybdenum loading, the 250-800-2h catalyst shows the highest catalytic activity with maximal exposure to active sites.

**Figure 5:**
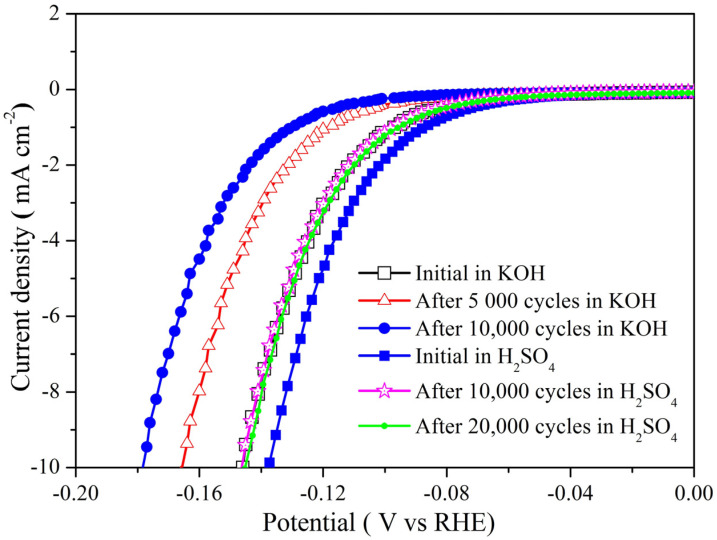
The catalytic stability test of the 250-800-2h catalyst in 0.5 M H_2_SO_4_ and 1 M KOH solution.

The catalytic stability of the 250-800-2h catalyst was evaluated under both acidic and alkaline conditions. As shown in Figure 5, the η_10_ of the catalyst is 137 mV at the first cycle in the acidic solution, and it increases to 145 mV after 10,000 cycles, then the η_10_ remains at 145 mV over 20,000 cycles. Moreover, the 250-800-2h catalyst could sustain the high catalytic activity during the 50 h stability test by chronoamperometry at −150 mV (Figure S5). This outcome suggests that the 250-800-2h catalyst has outstanding catalytic stability under acidic conditions. In contrast, the η_10_ of the 250-800-2h catalyst increases from 145 mV to 165 mV within 5000 cycles and comes to 177 mV after 10,000 cycles in the alkaline solution. This continuously slow increase in η_10_ implies the gradual attenuation of catalytic activity. Considering that molybdenum itself is alkali-soluble, the slow fade of catalytic activity can be attributed to the dissolution loss of molybdenum. Although the electrocatalytic HER activity and stability of the 250-800-2h catalyst in an alkaline system is not as good as that in an acidic medium, such performance is quite competitive with that of most carbon-supported molybdenum carbide materials reported recently (Table S1).

The high catalytic activity of the 250-800-2h catalyst is closely related to the synergistic effect of the components. The surface amino groups of CNTs tend to bind molybdenum atoms that help to make compact connections between Mo_2_C particles and CNTs, and the high electron-conductive CNTs can contribute greatly to enhanced catalytic reaction kinetics.

**Figure 6:**
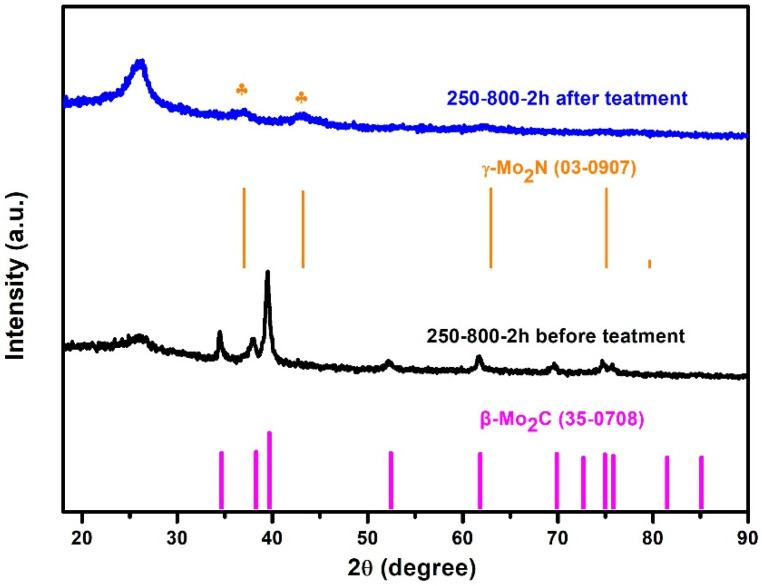
XRD patterns of the 250-800-2h catalyst before and after acid treatment.

The superior catalytic stability of the 250-800-2h catalyst in an acidic system was further explored. The active corrosion experiment was carried out by acid treatment of the 250-800-2h sample for 72 h. It is observed in Figure 6 that the main characteristic diffraction peaks of β-Mo_2_C disappear after acid treatment. This suggests that the surface β-Mo_2_C nanocrystals of the catalyst have been subjected to acid corrosion. There are two new weak peaks at 37.28° and 43.47°, corresponding to (111) and (200) planes of γ-Mo_2_N, respectively. It is speculated that the occurrence of γ-Mo_2_N characteristic peaks is the result of the corrosion of surface β-Mo_2_C crystals and more exposure to γ-Mo_2_N planes. The mass content of Mo in the 250-800-2h sample before and after acid treatment was analyzed by using TG. Considering that the final product in the oxygen atmosphere is MoO_3_ during TG measurements, the mass content of Mo (W_(Mo)_) in the raw material can be calculated by the formula:

$$W_{\left(\mathrm{Mo}\right)}{=W}_{{(\mathrm{MoO}}_{3})}{\;\cdot \;M}_{\left(\mathrm{Mo}\right)}{\;/\;M}_{{(\mathrm{MoO}}_{3})}$$

**Figure 7:**
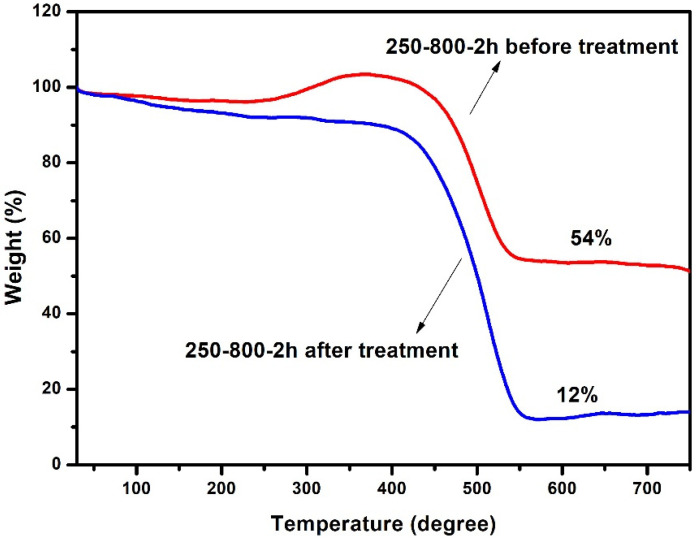
TG curves of the 250-800-2h catalyst before and after acid treatment.

The TG curves are shown in Figure 7. After the acid treatment, the final $W_{{(\mathrm{MoO}}_{3})}$ was decreased from 54% to 12%; that is to say, the total mass content of Mo in the catalyst was reduced by 78%. This actually explains why the XRD diffraction peaks of Mo_2_N cannot be observed in the primary 250-800-2h sample.

**Figure 8:**
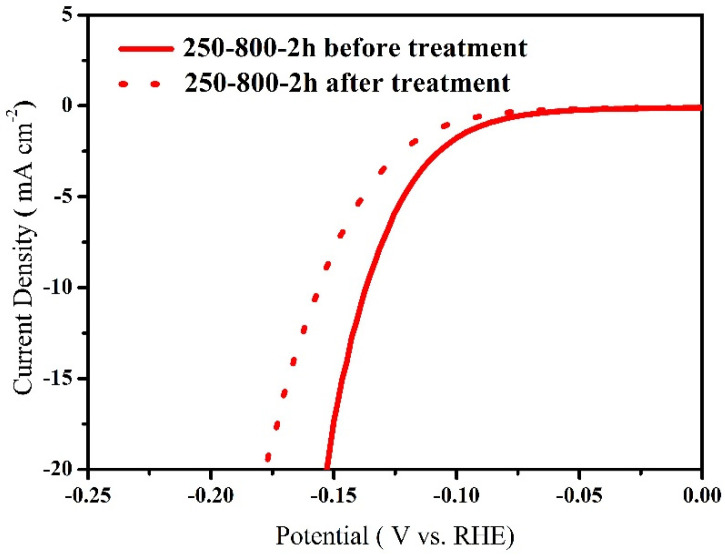
Polarization curves of the 250-800-2h catalyst before and after acid treatment in 0.5 M H_2_SO_4_ solution.

Figure 8 compares the catalytic activity of the 250-800-2h catalyst before and after acid treatment. It can be seen that the total catalytic activity is decreased after acid treatment. But taking into account the Mo content, the specific mass activity after acid treatment is 1.9 times that before acid treatment at 150 mV overpotential. This result hints that the catalytic utilization of Mo is greatly improved after acid corrosion. In fact, during the electrochemical stability test, the 250-800-2h catalyst is under the reductive atmosphere, which has far less corrosion effect on the catalyst than the active corrosion experiment. Therefore, the active corrosion test results can well explain and predict the high catalytic stability of the 250-800-2h catalyst under acidic conditions.

## 4. Conclusions

The CNT-supported molybdenum carbide HER catalysts have been fabricated using ammonium molybdate, amino CNTs, and methane as the precursors. The obtained 250-800-2h catalyst showed excellent catalytic performance in both acidic and alkaline solutions. Under the acidic condition, the η_10_ and Tafel slopes were 137 mV and 49.9 mV dec^−1^, respectively. The η_10_ decreased only 8 mV after 20,000 cycles of voltammetry scanning in an acidic solution. The corrosion test showed that the Mo content of the 250-800-2h catalyst could decrease by 78%, but its specific Mo mass activity actually increased by 1.9 times. The excellent performance of the catalyst was attributed to the synergistic effect of uniform distribution of molybdenum carbide on the surface of CNTs, the strong binding between molybdenum carbide and CNTs, the electronic modification of N doping, and protection of the carbon layer on the surface of molybdenum carbide. This phenomenon of increased activity accompanied by corrosion further provides a theoretical basis for its good activity durability in the actual use process.

## Data Availability

The data presented in this study are available on request from the corresponding author.

## Supplementary Materials

The following supporting information can be downloaded at: https://www.mdpi.com/article/10.3390/molecules28010192/s1, Figure S1: Calibration of SCE vs. RHE; Figure S2: SEM image and corresponding elemental distributions; Figure S3: (a) Polarization curves and (b) capacitive plots at 0.15 V of catalysts in 0.5 M H_2_SO_4_ solution; Figure S4: (a) Polarization curves and (b) capacitive plots at 0.15 V of catalysts in 0.5 M H_2_SO_4_ solution; Figure S5: Catalytic stability test of 250-800-2h catalyst at −0.15 V in 0.5 M H_2_SO_4_ solution; Table S1: Comparison of the catalytic performance for HER between carbon-supported molybdenum carbide of this work and reported recently. References [32,33,34,35,36,37,38,39,40,41] are cited in the supplementary materials.

## Sample Availability

Samples of the compounds are not available from the authors.
