# Supplementary material for: Carbon Nanotube Supported Molybdenum Carbide as Robust Electrocatalyst for Efficient Hydrogen Evolution Reaction
Source: Molecules. 2022 Dec 26;28(1):192. doi: 10.3390/molecules28010192 (PMC9822247; doi:10.3390/molecules28010192)
Supplement: Supplementary file 1 [file molecules-28-00192-s001.zip › molecules-2090850-supplementary.pdf]

Carbon Nanotube Supported Molybdenum Carbide as Robust Electrocatalyst for Efficient Hydrogen Evolution Reaction

Yunjie Huang [1], Yaqi Bao [1], Tieqi Huang [2], Chengzhi Hu [1], Haiou Qiu [1], Hongtao Liu [2,*]

[1] Faculty of Materials Science and Chemistry, China University of Geosciences, Wuhan 430074, China

[2] College of Chemistry and Chemical Engineering, Central South University, Changsha 410083, China

*** Correspondance:** liuht@csu.edu.cn

Table S1 Comparison of the catalytic performance for HER between carbon supported molybdenum carbide of this work and reported recently

Catalyst	η_{10} (mV)	Tafel slope (mV dec^{-1})	CV cycles for stability test	CV potential range for stability test (V)	Electrolyte	Reference
Mo_2C/N-CNTs	137	50	20000	-0.3~0.3	0.5M H_2SO_4	This work
	145	47	10000	-0.3~0.3	1M KOH	
Mo_2C/CNT-RGO	145	64	1000	-0.3~0.2	0.5M H_2SO_4	1
N-Mo_xC@C HSs	172	60	\	\	0.5M H_2SO_4	2
Co/Mo_2C@N-CNTs	170	92	1000	\	1M KOH	3
Mo_2C/CNTs	255	103	1000	\	0.5M H_2SO_4	4
MoC_x/N-CNTs	136	56	3000	-0.4~0	0.5M H_2SO_4	5
N,P-Mo_xC/NF	107	65	1000	-0.2~0.2	0.5M H_2SO_4	6
	135	57	1000	\	1M KOH	
Mo_2C/CNTs	110	51	1000	\	0.5M H_2SO_4	7
Mo_2N -Mo_2C/HGr	157	55	2000	\	0.5M H_2SO_4	8
	154	68	2000	\	1M KOH	
Mo_2C@N-DC/G	107	66	1000	0.17~0.27	0.5M H_2SO_4	9
Mo_2C/Carbon cloth	72	53	5000	-0.3~0	1M KOH	10
Mo_2C/CNTs	112	54	1000	\	0.5M H_2SO_4	11

Mo_2C/CNT-RGO	200	67	\	\	0.5M H_2SO_4	12
Mo_2C/NC	152	58	\	\	0.5M H_2SO_4	13
	135	56	1000	-0.6~0	1M KOH	
Mo_2C/N-CNTs	195	75	1000	\	0.5M H_2SO_4	14
Mo_2C/B,N-C	184	68	3000	\	0.5M H_2SO_4	15
	145	57	3000	\	1M KOH	
Mo_2C/NC	187		1000	\	0.5M H_2SO_4	16
	116	48	1000	\	1M KOH	
N,P-Mo_2C/C	103	57	2000	-0.4~0.05	0.5M H_2SO_4	17
	80	46	\	\	1M KOH	

Reference

1. Lee, G. H.; Lee, M. H.; Kim, Y.; Lim, H. K.; Youn, D. H., Facile synthesis of nanostructured molybdenum carbide/nitrogen-doped CNT-RGO composite via a modified urea glass route for efficient hydrogen evolution. *J. Alloys Compd.* **2019,** *805*, 113-119.
2. Xiong, T.; Jia, J.; Wei, Z.; Zeng, L.; Deng, Y.; Zhou, W.; Chen, S., N-doped carbon-wrapped Mo C heterophase sheets for high-efficiency electrochemical hydrogen production. *Chem. Eng. J.* **2019,** *358*, 362-368.
3. Ouyang, T.; Ye, Y. Q.; Wu, C. Y.; Xiao, K.; Liu, Z. Q., Heterostructures composed of n-doped carbon nanotubes encapsulating cobalt and beta-Mo_2c nanoparticles as bifunctional electrodes for water splitting. *Angew. Chem. Int. Ed.* **2019,** *58* (15), 4923-4928.
4. Pu, J. X.; Cao, J.; Ma, L.; Zhou, K. C.; Yu, Z. M.; Yin, D. F.; Wei, Q. P., Novel three-dimensional Mo_2C/carbon nanotubes composites for hydrogen evolution reaction. *Mater. Lett.* **2020,** *277*.
5. Jo, H. M.; Kim, Y.; Youn, D. H., One-pot synthesis of molybdenum carbide/N-doped carbon nanotube composite using nitrilotriacetic acid for efficient hydrogen evolution. *J. Alloys Compd.* **2021,** *855*.
6. Ji, L.; Wang, J.; Teng, X.; Dong, H.; He, X.; Chen, Z., N,P-Doped molybdenum carbide nanofibers for efficient hydrogen production. *Acs Appl. Mater. Interfaces* **2018,** *10* (17), 14632-14640.
7. Adam, A.; Suliman, M. H.; Awwad, M.; Siddiqui, M. N.; Yamani, Z. H.; Qamar, M., Controlled growth of small and uniformly dispersed Mo_2C on carbon nanotubes as high performance electrocatalyst for the hydrogen evolution reaction. *Int. J. Hydrogen Energy* **2019,** *44* (23), 11797-11807.
8. Yan, H.; Xie, Y.; Jiao, Y.; Wu, A.; Tian, C.; Zhang, X.; Wang, L.; Fu, H., Holey reduced graphene oxide coupled with an Mo_2N-Mo_2C heterojunction for efficient hydrogen evolution. *Adv. Mater.* **2018,** *30,* 29164704.

9. Wang, B.; Wu, X. Y.; Zhang, X. Y.; Pang, G. G.; Li, S. M., Mo_2C-embedded biomass-derived honeycomb-like nitrogen-doped carbon nanosheet/graphene aerogel films for highly efficient electrocatalytic hydrogen evolution. *New J. Chem.* **2020,** *44* (3), 1147-1156.
10. He, M. C.; Shi, H. Y.; Wang, P.; Sun, X. D.; Gao, B., Porous Molybdenum carbide nanostructures synthesized on carbon cloth by CVD for efficient hydrogen production. *Chem. Eur. J.* **2019,** *25* (70), 16106-16113.
11. Hu, Y.; Guan, D. G.; Yu, B.; Hou, W. Q.; Zheng, B. J.; Zhang, W. L.; Chen, Y. F., Scalable synthesis of Mo_2C/CNT networks as highly efficient and stable electrocatalyst for hydrogen evolution reaction. *Electrochim. Acta* **2018,** *263*, 192-200.
12. Lee, Y. H.; Brahma, S.; Huang, P. C.; Wang, S. C.; Huang, J. L., Molybdenum carbide (Mo_2C) and reduced graphene oxide (rGO) nano-composites as an efficient electrocatalyst for water splitting. *Mater. Lett.* **2022,** *316,* 131934.
13. Wang, J.; Li, S. W.; Hu, J.; Niu, S. Q.; Li, Y. Z.; Xu, P., Acid-directed morphology control of molybdenum carbide embedded in a nitrogen doped carbon matrix for enhanced electrocatalytic hydrogen evolution. *Inorg. Chem. Front.* **2020,** *7* (19), 3620-3626.
14. Song, Y. J.; Ren, J. T.; Yuan, G. G.; Yao, Y. L.; Liu, X. Y.; Yuan, Z. Y., Facile synthesis of Mo_2C nanoparticles on N-doped carbon nanotubes with enhanced electrocatalytic activity for hydrogen evolution and oxygen reduction reactions. *J. Energy Chem.* **2019,** *38*, 68-77.
15. Wu, S. F.; Chen, M. Y.; Wang, W. W.; Zhou, J. B.; Tang, X. R.; Zhou, D. L.; Liu, C., Molybdenum carbide nanoparticles assembling in diverse heteroatoms doped carbon matrix as efficient hydrogen evolution electrocatalysts in acidic and alkaline medium. *Carbon* **2021,** *171*, 385-394.
16. Wang, S. Q.; Li, Y. Z.; Xie, J.; Hao, A. Z.; Cao, Y. L., One-pot solution-free construction for hybrids of molybdenum carbide nanoparticles and porous N-doped carbon nanoplates as efficient electrocatalyst of hydrogen evolution. *J. Alloys Compd.* **2021,** *861,*157935.

17. Wang, J. H.; Wei, H. F.; Chen, X.; Chen, C. S.; Chen, X. A., Facile preparation of N, P co-doped molybdenum carbide/porous carbon rough microspheres for efficient electrocatalytic hydrogen evolution. *Int. J. Hydrogen Energy* **2020,** *45* (1), 595-604.

Table 1 e Comparison of the catalytic activity for HER between NP-Mo_2C/PCMS-2-36-750 and other biomass-derived carbon/Mo_2C and several Mo_2C-based catalysts reported recently.

The calibration of SCE reference electrode was tested in a standard three-electrode system with two polished Pt electrodes and our SCE as the working, counter electrodes and reference electrode, respectively. Electrolytes were pre-purged high purity hydrogen to form a saturated solution of hydrogen. Then, linear scanning voltammetry (LSV) was performed at a scan rate of 0.1 mV/s. The potential at the current of zero was considered as the thermodynamic potential (vs. SCE) and was found to be -0.262 V and -1.04 V in 0.5 M H_2SO_4 and 1 M KOH solution, respectively.

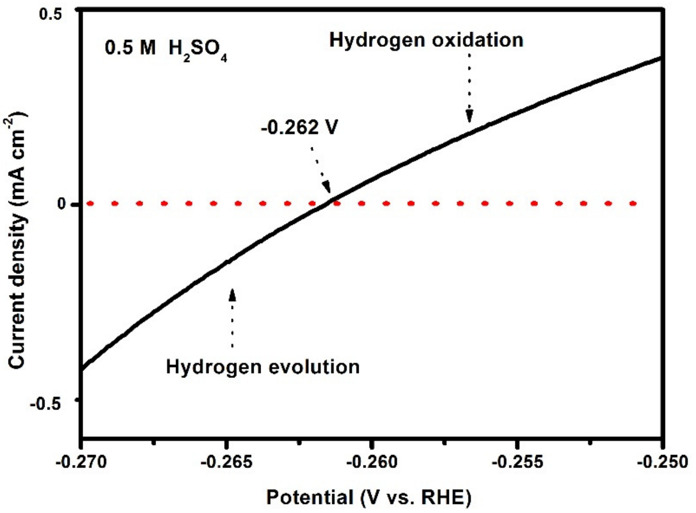

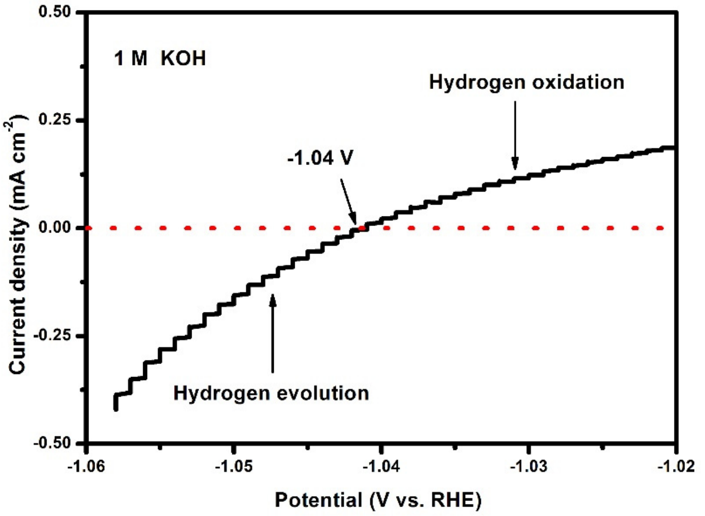

Figure S1 Calibration of SCE vs RHE.

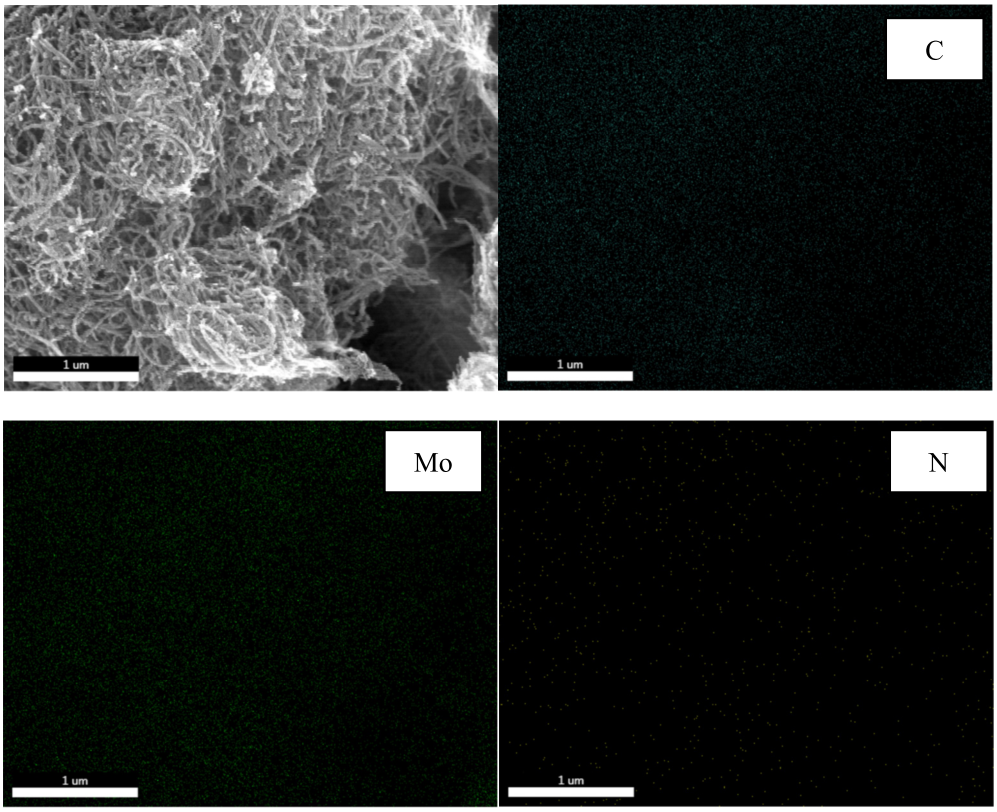

Figure S2 SEM image and corresponding elemental distributions.

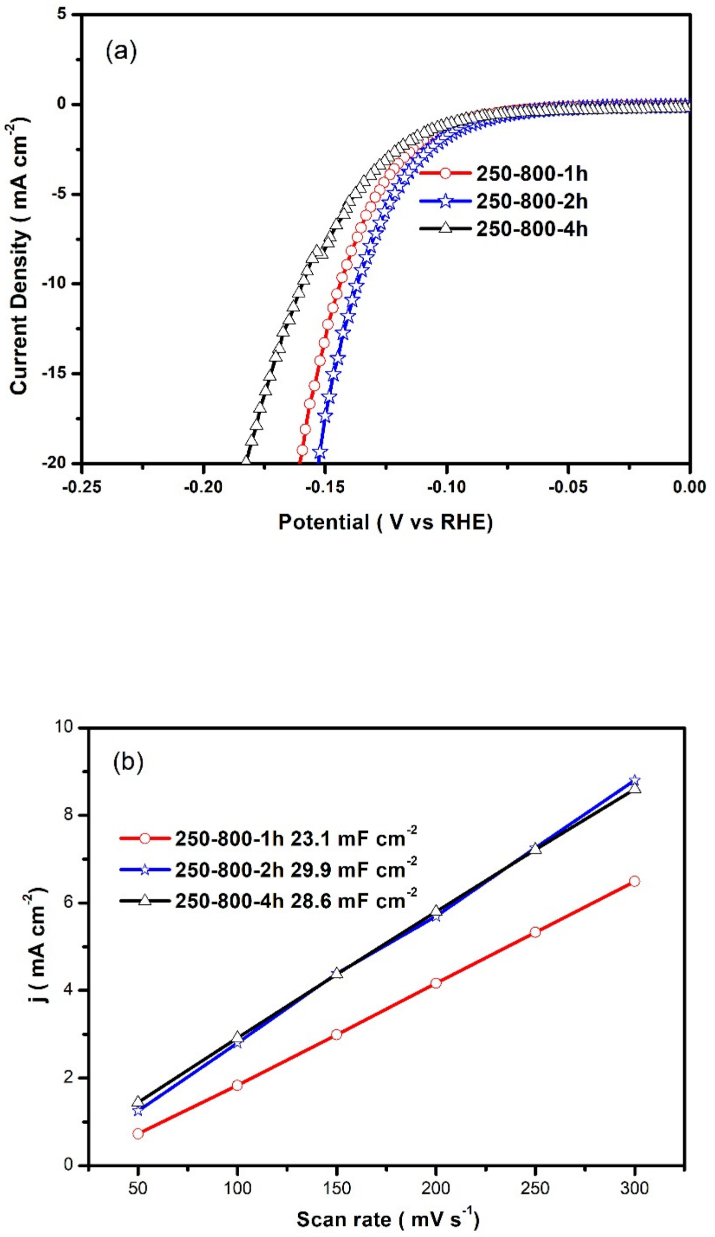

Figure S3 (a) polarization curves and (b) capacitive plots at 0.15 V of catalysts in 0.5 M H_2SO_4 solution.

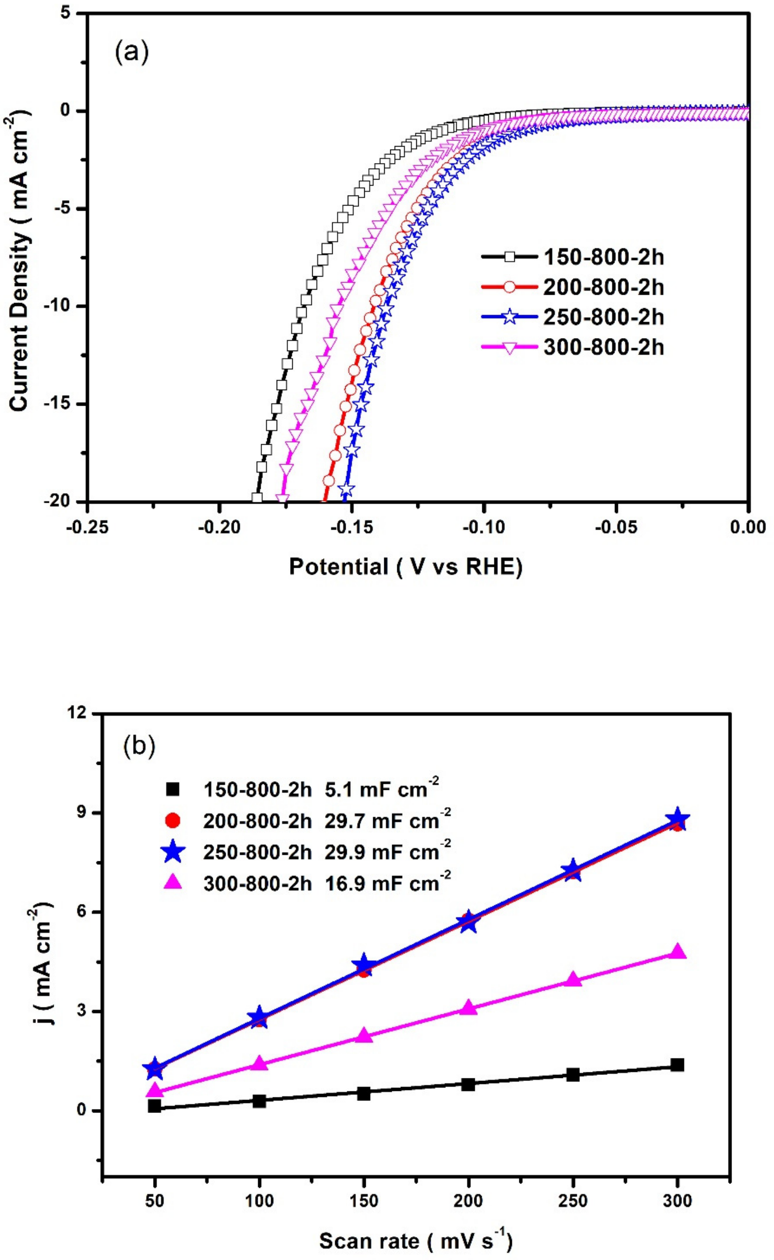

Figure S4 (a) polarization curves and (b) capacitive plots at 0.15 V of catalysts in 0.5 M H_2SO_4 solution.

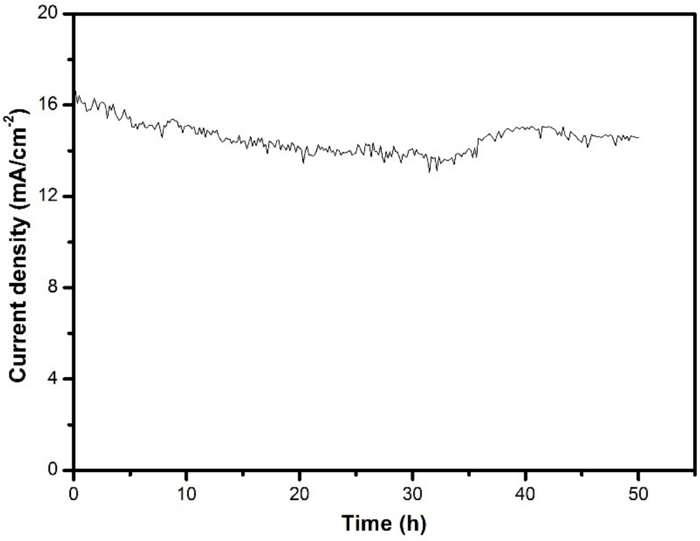

Figure S5 Time dependence of current density over 250-800-2h catalyst during HER at -0.15 V in 0.5 M H_2SO_4 solution
